# Adolescent mental health and cardiorespiratory fitness: A comparison of two cohorts 12 years apart

**DOI:** 10.1371/journal.pone.0300810

**Published:** 2024-05-15

**Authors:** Ottar Birgisson, Hege R. Eriksen, Mari Hysing, Erlingur Johannsson, Sunna Gestsdottir

**Affiliations:** 1 Center of Sport and Health Sciences, School of Education, University of Iceland, Reykjavik, Iceland; 2 Department of Sport, Food and Natural Sciences, Western Norway University of Applied Sciences, Bergen, Norway; 3 Faculty of Psychology, Department of Psychosocial Science, University of Bergen, Bergen, Norway; Bangor University, UNITED KINGDOM

## Abstract

The aim of the study was to compare the mental health and cardiorespiratory fitness (CRF) of adolescents in two cross-sectional cohorts, one measured in 2003 and the other in 2015, both at age 15 and across sexes. The study also sought to estimate the association between mental health and CRF in the two cohorts and examine the relationship between the level of CRF and mental health in each cohort overall and by sex. Data from 443 participants born in 1988 (228 males, 215 females) and 303 participants born in 1999 (126 males, 177 females) were analyzed. Mental health was assessed using self-reports of body image, self-esteem, and symptoms of depression and anxiety. CRF was estimated using a maximal cycle ergometer test. From 2003 to 2015, body image scores improved (p = .043), self-esteem remained stable, and CRF declined significantly (p < .001). No self-esteem differences were observed between sexes in any cohort. Males had higher CRF and body image scores than females in both cohorts (p < .001 for all comparisons). Higher CRF correlated with fewer depressive symptoms across sexes and cohorts. Specifically, higher CRF was associated with anxiety in females and improved body image in males (2003) and both sexes (2015). Increased CRF was linked to higher self-esteem in females but not in males. Overall, higher CRF levels were associated with better mental health outcomes for both sexes. These results highlight the potential of improving adolescent mental health through increased physical fitness.

## Introduction

Adolescence is marked by profound physical, social, and psychological transformations. Recent trends suggest that both mental health and cardiorespiratory fitness (CRF), which is a marker for physical health, are declining over time [1–4], with a potential link between the two [5]. Notably, rates of depression have increased among adolescents [6,7], particularly among females [8,9]. Moreover, low self-esteem, depression, and anxiety tend to be more prevalent among females than males, during adolescence and adulthood [10–13]. Body image dissatisfaction is getting worse for adolescents and is more prevalent with girls and women [14]. When it comes to sex differences in CRF, males tend to have higher levels than females [15,16]. However, longitudinal studies are lacking when it comes to assessing if males and females show different trends in CRF.

The relationship between CRF and mental health is complex. Studies indicate that lower CRF increases the risk of mental health problems [5] and conversely, higher CRF is linked to more positive mental health outcomes [17,18]. Furthermore, intervention programs targeting CRF have shown promise in enhancing self-esteem [19,20]. Additionally, high CRF has also been shown to predict more positive body image longitudinally [21].

Overall, these trends point to a concerning scenario in which both physical and mental health among adolescents appear to be deteriorating. Thus, it is of interest to examine the changes in mental health and CRF and the relationship between the two at the beginning of the 21st century in Iceland.

In our study, results from two 15 years old Icelandic cohorts, one born in 1988 and the second born in 1999 were compared. Note, that in a recently published article using the same data, we investigated the comparasion between depressive sypmtoms and anxiety where we discovered that depressive symptoms are worse for females in the 1999 cohort then the 1988 cohort. [8]. Therefore, the objective of this study was twofold.

Firstly, to conduct a comparative analysis of body image, and self-esteem, and CRF in these two cohorts aged 15. Secondly, to assess the association between mental health (symptoms of depression, symptoms of anxiety, body image, and self-esteem) and CRF, in both cohorts across sexes. In relation to the second aim, the relationship will also be assessed between three levels of CRF (low, medium, and high) across the mental health variables.

## Materials and method

### Design and subjects

**Fig 1:**
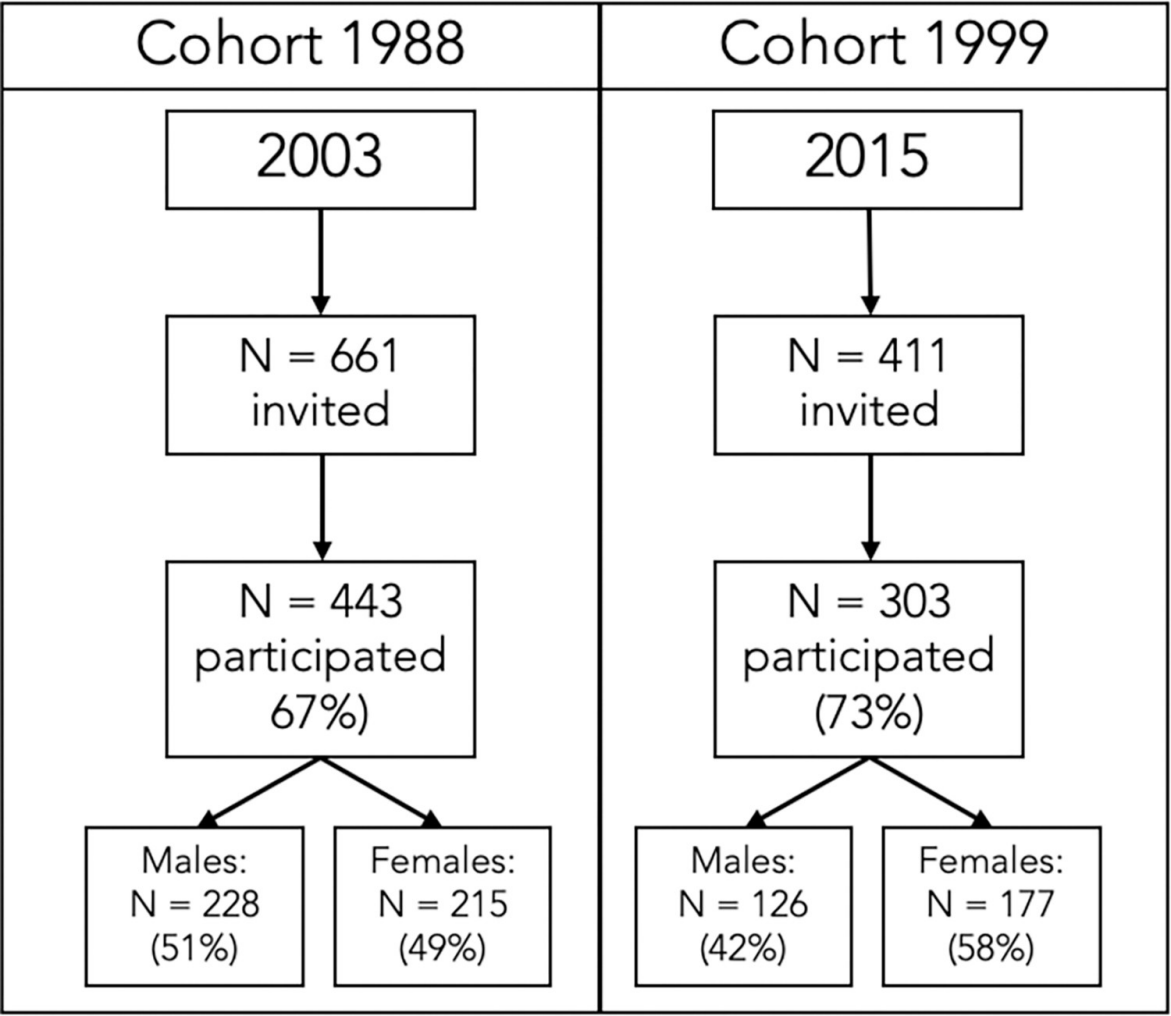
The attrition of the participants used in the study. Figure shows total participation while participation between measures varied.

Data came from two longitudinal cohort studies, one cohort born in 1988 (n = 443) and the other cohort in 1999 (n = 303) both measured at age 15. A total of 746 (392 females and 354 males) participated. See attrition chart in Fig 1. Data from the 1988 cohort [22] was collected between August 2003 and January 2004, and the participants came from 18 randomly selected schools from different parts of Iceland, in proportion to where the population lived (60% from the capital area, 35% other urban area, and 5% rural area). Every student in 10^th^ grade from the selected schools were offered to participate through the school and on school time. The 1999 cohort came from six randomly selected schools in the capital city and was measured between January and March 2015. All 10^th^ graders in those schools were offered to participate through the school and on school time. In both cohorts, the primary reasons for not participating were being absent from school on the days of measurement and possibly having no interest in the study.

Because of the discrepancy of location of participants between the years, the data from 2003 was split into two distinct groups: Group 1 from the capital area and Group 2 from regions outside the capital. Analysis of the key variables in our study revealed no significant differences between participants from these two groups giving evidence that there is little or no difference between participants from the capital area and other locations. Further description of the invitation to participate in the studies, participation rate, and dropout, has been published already. See Johannsson et al. (2006) [22], and Gestsdottir et al. (2015) [23] for the 1988- cohort, and Brychta et al. (2019) [24] for the 1999-cohort, and Birgisson et al. (2023) for both cohorts. Access to data for this retrospective study was granted and accessed on October 1^st^ 2021 with no identifiable information.

### Ethics

Both research projects were approved by the Icelandic Data Protection Authority according to the Icelandic Act on Processing of Personal Data, and the Icelandic Bioethics Committee (VSNb200605002/03 for 2003 and VSNb2015020013/13.07 for 2015). Written informed consent was obtained from all the participants and their parents before data collection, for details see Johannsson et al. (2006) [22] and Brychta et al. (2019) [24]. Strict procedures were followed to ensure confidentiality.

### Measures

In both cohorts, the same self-reported questionnaires in Icelandic were used to measure mental health, and the same objective measures were collected on physical health (CRF and BMI).

#### Socioeconomic status (SES)

Parents’ education was measured with questions about their education level, e.g., if they finished primary school, secondary school, apprenticeship, or university. Participants also answered questions about whom they lived with, e.g., with both parents, with either mother or father, with grandparents, or other living arrangements. To simplify analysis for the multiple regression one variable was created to indicate SES where the value of 3 was assigned to participants who had both parents with a university education and live with both parents, a value of 2 to participants who have either one parent with a university education or live with both parents and a value of 1 to participants who have neither parent with a university education nor live with both parents [25–27].

#### Depression

Symptoms of depression was measured with a ten-item Subscale of Symptom Checklist 90 (SCL-90)[28]. It was scored on a five-point Likert scale rated from 1 (almost never) to 5 (almost always), asking about feelings of depression like “*Did you feel that the future is hopeless*”, in the preceding week. Higher scores indicate more symptoms of depression. Cronbach’s alpha for the full scale was used to evaluate internal consistency, which was α = 0.88 (good reliability) for the 1988 cohort and α = 0.94 (excellent reliability) for the 1999 cohort.

#### Anxiety

Symptoms of anxiety was measured with a four-item Subscale of Symptom Checklist 90 (SCL-90)[28]. It was scored on a five-point Likert scale rated from 1 (almost never) to 5 (almost always), asking about feelings like ‘Suddenly scared for no reason’ in the preceding week. Higher scores indicate a higher level of anxiety. Cronbach’s alpha for the full scale was used to evaluate internal consistency, which was α = 0.74 (acceptable reliabililty) for the 1988 cohort and α = 0.88 (good reliability) for the 1999 cohort.

#### Body Image

Body image was assessed with five items from the *Body and Self-Image subscale of the Offer Self-Image Questionnaire* [29]. Participants were asked how well they agreed with five statements e.g. “*I’m satisfied when I think about how my body will look in the future*“. All items were rated on a four-point response scale, where *1 = Not at all true of me*, and *4 = True of me*. Higher scores indicate higher levels of body image (i.e. more positive body image). Cronbach’s alpha for the scale was used to evaluate internal consistency, which was α = 0.70 (acceptable reliability) for the 1988 cohort and α = 0.85 (good reliability) for the 1999 cohort.

#### Self-esteem

Global self-esteem was assessed, using the Rosenberg Self-Esteem Scale [30,31]. The scale consists of ten statements, each rated as negative or positive, with four response options ranging from “strongly agree”(3) to “strongly disagree”(0). Higher scores (15 points or higher) reflect a higher level of self-esteem. The Rosenberg scale has been widely used in measuring self-esteem in young people, and its reliability and validity are well documented [13,32]. Higher scores indicate a higher level of self-esteem. Cronbach’s alpha was used to evaluate the internal consistency of the scale, which was α = 0.87 (good reliability) for the 1988 cohort and α = 0.91 (excellent reliability) for the 1999 cohort.

#### Cardiorespiratory fitness

CRF was assessed objectively using the maximal cycle ergometer test on a Monark stationary bike [33]. CRF are in similar studies expressed as absolute maximal power output (W_max_) as well as maximal power output relative to body mass (W/kg) to account for differences in body size [34,35]. The mechanical power (W_max_) was used in this study, after being dived by the participants’ weight, to assume the VO_2_ max. The test has been validated in adolescents and predicted oxygen uptake from this test correlates very well with measured oxygen uptake [36,37]. Furthermore, Saevarsson et al. found no significant differences when CRF was expressed in relative or absolute terms, supporting the use of relative measure in this study [38]. However, to ensure that possible effects of CRF are not attributable to differences in participants weight we also included the absolute value (W_max_).

#### Body mass index

Body mass index (BMI) was calculated from participants´ measured weight and height (kg/m^2^).

#### Self-reported vigorous physical activity

The physical activity was assessed with the question “How often, per week, do you perform physical activity that makes you breathe more rapidly or sweat?”. Response options were six ranging from 1 = never to 6 = almost every day.

### Statistical analysis

Descriptive summaries are presented as means and standard deviations for continuous variables and as frequencies or percentages for categorical variables. Study variables were analyzed for distributional properties. The alpha level for significant differences is set at 0.05. To assess differences between cohorts an independent sample t-test was used when the variance was equal and Welch’s T-test when the variance was assumed unequal. Cohen’s *d* effect size represents the differences between cohorts for continuous variables and Cramer´s *V* for categorical. For the multiple linear regression analyses, our goal was to examine the relationship between mental health outcomes (as dependent variables) and CRF, adjusting for SES as a covariate. Specifically, the models were structured as follows: the dependent variable (Y) represented the mental health measures (body image, depressive symptoms, anxiety symptoms, and self-esteem), and the independent variables included CRF (W_max_ /kg) as X1 and SES (encompassing both parental education and living arrangement) as X2. The adjusted R-squared values were reported to quantify the proportion of variance in the mental health outcomes explained by the models, after accounting for the effects of SES. Finally, the CRF variable (W_max_ /kg) was divided into three levels by using cut points for three equal groups (Low levels: <2.65, Medium levels: 2.65–3.39, and High levels: >3.39). An analysis of variance (ANOVA) was used to assess if there was a difference between the level of CRF and body image, symptoms of depression and symptoms of anxiety, and self-esteem for both cohorts across the sexes.

## Results

### Demographic characteristics

Participants SES based on parents’ education and living arrangements changed from 2003 to 2015 (Table 1). In 2003 a higher proportion of participants lived with both parents (χ^2^ (4) = 21.04, p < .001, V = 0.18) and a higher proportion had a mother with university education (χ^2^ (3) = 97.40, p < .001, V = 0.44) than in 2015.

**Table 1:** Descriptive statistics for participants SES and weekly physical activity.

		2003		2015	
		Male	Female	Male	Female
Lives with	both parents	145 (74.4%)	129 (67.9%)	76 (61.3%)	90 (51.1%)
	mother only	21 (10.8%)	28 (14.7%)	15 (12.1%)	30 (17.0%)
	father only	8 (4.1%)	6 (42.9%)	15 (12.1%)	17 (9.7%)
	mother and stepparent	16 (18.2%)	22 (11.6)	9 (7.3%)	20 (11.4%)
	father and stepparent	1 (0.5%)	3 (1.6%)	3 (2.4%)	4 (2.3%)
	other arrangements	3 (1.5%)	2 (1.1%)	6 (28.6%)	15 (71.4%)
	Total	195 (100%)	190 (100%)	124 (100%)	176 (100%)
Mother‘s education	University	42 (37.5%)	50 (40.3%)	84 (72.4%)	98 (60.1%)
	Apprenticeship	8 (7.1%)	4 (3.2%)	17 (14.7%)	35 (21.7%)
	Secondary school	25 (22.3%)	27 (21.8%)	4 (3.4%)	15 (9.3%)
	Primary school	37 (33.0%)	43 (34.7%)	11 (9.5%)	13 (8.1%)
	Total	112 (100%)	124 (100%)	116 (100%)	161 (100%)
Father‘s education	University	27 (27.0%)	40 (36.4%)	58 (49.2%)	86 (57.7%)
	Apprenticeship	40 (40.0%)	31 (28.2%)	15 (12.7%)	15 (10.1%)
	Secondary school	15 (15.0%)	11 (10.0%)	33 (28.5%)	33 (22.1%)
	Primary school	18 (18.0%)	28 (25.5%)	12 (10.2%)	15 (10.1%)
	Total	100 (100%)	110 (100%)	118 (100%)	149 (100%
Physical activity^a^	Never	8 (4.1%)	4 (2.1%)	0 (0.0%)	6 (3.5%)
	Less than 1x a week	8 (4.1%)	15 (7.9%)	5 (4%)	11 (5.4%)
	1x a week	13 (6.7%)	22 (11.6%)	9 (7.3%)	17 (9.8%)
	2-3x a week	34 (17.5%)	51 (27%)	24 (19.4%)	42 (24.3%)
	4-5x a week	60 (30.9%)	60 (31.7%)	37 (29.8%)	47 (27.2%)
	Almost every day	71 (36.6%)	37 (19.6%)	49 (39.5%)	50 (28.9%)
	Total	194 (100%)	189 (100%)	124 (100%)	173 (100%)

^a^ Self-reported vigorous physical activity.

### Differences between the 1988 and 1999 cohorts

The body image score was higher in 2015 (t(670) = -2.03, p = .043) than in 2003. Self-esteem remained stable over the years. These findings are further detailed in Table 2. In 2003 CRF (relative values) scores (t(504) = 10.73, p < .001) as well as absolute values (t(523) = 8.59, p < .001) were significantly higher and BMI (t(751) = -4.26, p < .001) was lower compared to scores in 2015 (Table 2). No change was found in self-reported vigorous physical activity (Table 1).

**Table 2:** Differences between 2003 and 2015 with 95% Confidence Intervals and effect sizes.

	M						95% CI	
	2003	2015	t-value	df	p-value	Effect Size	Lower	Upper
BMI	21.03	22.00	-4.26	751	< .001	0.32	-0.46	-.017
CRF (W/kg)	3.42	2.80	10.73	504	< .001	0.96	0.76	1.15
W_max_	210	166	8.59	523	< .001	0.73	0.55	0.91
Self-Esteem	31.47	31.31	0.32	666	.752	0.03	-0.13	0.18
Body-Image	14.85	15.33	-2.03	670	.043	0.16	-0.31	-0.01

Note. BMI refers to Body Mass Index. CRF = Cardiorespiratory fitness(W/kg), relative values, W_max_ = Absolute values of CRF, M = Mean score. Df = Degrees of freedom. Effect sizes are reported as Cohen’s d. CI = Confidence Interval.

### Differences between the sexes

In 2003, males had higher CRF, self-esteem, and body image (t(222) = 11.34, p < .001; t(369) = 2.30, p = .022; t(372) = 4.82, p < .001, respectively) compared to females. No sex-based difference was observed in 2003 regarding BMI.

In 2015, males demonstrated higher CRF (t(291) = 8.38, p < .001), higher self-esteem (t(295) = 2.40, p = .017), and higher body image scores (t(287) = 5.65, p < .001). However, there was no significant difference in BMI between sexes in 2015 (Table 3).

**Table 3:** Differences between the sexes for 2003 and 2015.

								95% CI	
	Year	Male	Female	t-value	df	p-value	Effect Size	Lower	Upper
BMI	2003	20.98	21.07	-0.32	441	.751	0.24	-0.65	0.47
	2015	21.74	22.18	-1.18	308	.239	0.14	-1.18	0.29
Cardiorespiratory fitness (W/kg)	2003	3.78	3.03	11.34	222	< .001	1.47	0.62	0.88
	2015	3.09	2.21	8.38	291	< .001	0.99	0.67	1.09
Cardiorespiratory fitness W_max_	2003	245.20	172.54	-16.27	216	< .001	2.09	-81.46	-63.85
	2015	209.80	134.60	-10.65	215	< .001	1.32	-89.11	-61.27
Self-Esteem	2003	32.15	30.75	2.30	369	.022	0.24	0.20	2.60
	2015	32.42	30.51	2.40	295	.017	0.28	0.34	3.47
Body-Image	2003	15.53	14.16	4.82	372	< .001	0.50	0.81	1.93
	2015	16.50	14.49	5.65	287	< .001	0.66	1.31	2.71

Note. BMI = Body Mass Index, df = Degrees of freedom. Effect size is Cohen’s d.

### Association between mental health and cardiorespiratory fitness

Between symptoms of depression and CRF, an association was observed for both males and females after adjusting for SES (see Table 4). Among males, CRF was significantly associated with symptoms of depression in 2003 (B = -3.83, SE = 1.42, p = 0.009) and 2015 (B = -1.23, SE = 0.59, p = 0.039). Similarly, among females, significant associations were found in 2003 (B = -4.87, SE = 1.92, p = 0.014) and 2015 (B = -2.27, SE = 1.99, p = 0.049).

**Table 4:** Association between mental health (symptoms of depression, anxiety, body image and self-esteem) on CRF after adjusting for SES.

	Year	Sex	R^2^	B	SE	p-value
CRF-> Depression	2003	Male	0.24	-3.83	1.42	0.009**
	2015	Male	0.10	-1.23	0.59	0.039*
	2003	Female	0.09	-4.87	1.92	0.014*
	2015	Female	0.06	-2.27	-1.99	0.049*
CRF -> Anxiety	2003	Male	0.04	-0.62	0.51	0.224
	2015	Male	0.09	-0.41	0.22	0.071
	2003	Female	0.06	-1.77	0.85	0.042*
	2015	Female	0.06	-1.14	0.46	0.014*
CRF-> Body Image	2003	Male	0.16	2.03	0.68	0.004**
	2015	Male	0.03	0.33	0.27	0.227
	2003	Female	0.14	2.39	0.75	0.002**
	2015	Female	0.06	0.92	0.36	0.012*
CRF -> Self-Esteem	2003	Male	0.12	2.24	1.28	0.087
	2015	Male	0.02	0.84	0.68	0.217
	2003	Female	0.10	3.87	1.65	0.022*
	2015	Female	0.06	1.41	0.97	0.019*

CRF = cardiorespiratory fitness (W/kg) and SES = socioeconomic status.

* p < 0.05

** p < 0.01.

Regarding the relationship between CRF and symptoms of anxiety, an association was found only among females, both in 2003 (B = -1.77, SE = 0.85, p = 0.042) and in 2015 (B = -1.14, SE = 0.46, p = 0.014).

When examining the relationship between CRF and body image, association was observed among males (B = 2.03, SE = 0.68, p = 0.004) and females in 2003 (B = 2.39, SE = 0.75, p = 0.002), but only females in 2015 (B = 0.92, SE = 0.36, p = 0.012).

Lastly, in the analysis of CRF and self-esteem, an association was found only among females both in 2003 (B = 3.87, SE = 1.65, p = 0.022) and 2015 (B = 1.41, SE = 0.97, p = 0.019).

### Differences in mental health depending on the level of cardiorespiratory fitness

**Fig 2:**
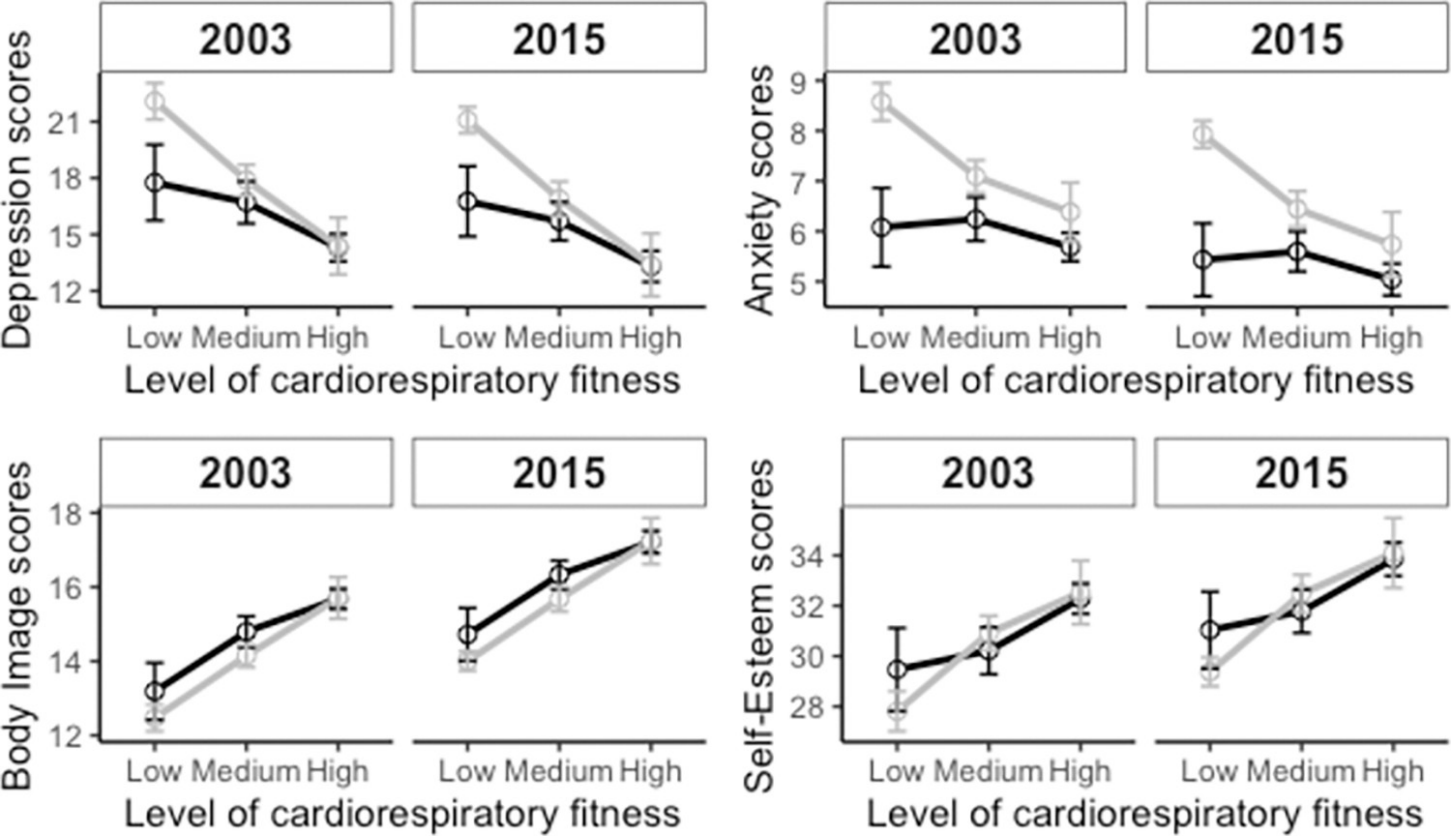
The relationship between three levels of cardiorespiratory fitness and symptoms of depression, anxiety, body image, and self-esteem score. Each part of the figure shows both the results for 2003 and 2015 with the black line indicating males and gray indicating females. Error bars show the standard error.

The analysis consistently demonstrated a significant main effect of CRF levels on mental health outcomes across all variables. Specifically, there was a significant reduction in depression (F(2, 459) = 29.254, p < 0.001) and anxiety scores (F(2, 461) = 20.000, p < 0.001), along with increases in body image (F(2, 458) = 26.113, p < 0.001) and self-esteem (F(2, 454) = 12.061, p < 0.001) associated with higher CRF levels. These effects were consistent across both the 2003 and 2015 measures, as depicted in Fig 2. Notably, no significant interaction effects between CRF levels and sex or year were found, indicating the relationship between CRF and mental health outcomes was stable irrespective of these variables.

## Discussion

### Main findings

In comparing 2015 to 2003, a decline in CRF was evident, while there was an improvement in body image. However, self-esteem showed no significant changes between these two cohorts. Males had higher self-esteem both in 2003 and 2015 though effect size was low. Furthermore, CRF and body image displayed consistent variations between sexes in both years, with males consistently exhibiting higher levels of CRF and more positive body image perceptions compared to females. Regarding the relationship between CRF and mental health, an association with symptoms of depression was observed for both males and females in both 2003 and 2015. However, the association between CRF and symptoms of anxiety was exclusively significant for females across both years, with no notable association found for males. Moreover, CRF was linked to body image for males in 2003 and for females in both 2003 and 2015, but this association was absent for males in 2015. Similarly, CRF was associated with self-esteem for females in both 2003 and 2015 but did not show a significant relation for males. In summary, these findings underscore a consistent relationship between higher CRF levels and improved mental health, with variations in how this connection manifests between sexes and across the years.

### Differences between the cohorts

Even though BMI got higher between the years the CRF got worse both in absolute and relative values, suggesting that the decline in CRF is independent of participants’ weight. The decline in CRF scores observed in this study can be understood in light of the low adherence of adolescents to the recommended levels of PA in the 2020 WHO guidelines on physical activity and sedentary behavior for children and adolescents aged 5–17 years [39]. Notably, the 2020 WHO guidelines acknowledge that PA and sedentary behavior are distinct entities according to new evidence, and therefore guidelines for sedentary behavior have now been included in the WHO guidelines [39]. This distinction may partly explain why the cohorts in this study report similar physical activity. Specifically, an increase in sedentary behavior may be a contributing factor in the increase in worse CRF, even though self-reported physical activity remains unchanged. However, as we did not have measures of sedentary behavior in this study these are merely speculations.

Females had worse CRF and worse body image than males both in 2003 and 2015. The sex differences regarding CRF are consistent with previous studies [40,41] as are the results on body image [21].

Self-Esteem appeared to be stable between 2003 and 2015 and self-esteem did not differ between sexes either year. The fact that there is no difference between sexes may be seen as surprising, however, a longitudinal study has shown that though males usually have higher self-esteem than females, it is least different at the age of 14 before it increases for males and decreases for females [42]. So far, there is no consensus on whether self-esteem is getting better or worse over time, and that could possibly be due to limitations of self-esteem measurements such as ceiling effects and skewed distribution of data [43].

The finding that CRF is getting worse suggests that there may be factors at play that are contributing to the decline for this age group. One such possible factor is, as mentioned above, the increased sedentary behavior with the emergence of screentime (specifically social media) with the former being known to affect physical and mental health [44] and the latter is negatively associated with mental health [8,45–48]. Sedentary behavior is related to less PA and studies have shown that PA is related to mental health [4,44,49] and it has been shown that an active lifestyle during childhood can have positive effects on mental health later in adolescence and life in general [50,51]. However, self-reported physical activity increased between years according to the current study. Furthermore, mediation analysis is needed to test these types of relationships. Another interesting finding of this current study is that despite the decline in CRF observed in the younger cohort, both cohorts reported relatively healthy body image and self-esteem.

### Association between CRF and mental health in each cohort

The present study revealed a significant association between CRF and symptoms of depression for both sexes in both years indicating that higher CRF could be important to consider when preventing or treating depression. In fact, a study has shown that interventions where the main focus is to improve CRF level can be effective as an alternative treatment for depression [52]. That study showed that CRF had more positive effect on depression then the control groups for strength training and relaxation [52]. However, the findings show a reduction in beta-coefficients from 2003 to 2015 for both males and females between CRF and depression. This means less explanatory power of CRF for variations in symptoms of depression between the years as seen by lower R^2^ from 2003 to 2015. One plausible interpretation of this trend could be the emergence and influence of new social and environmental factors that were less prevalent in 2003. For instance, the rise of social media and its impact on mental health [45] could be a contributing factor and potentially diluting the relative influence of traditional predictors like CRF.

According to the current results, there was only a relationship between symptoms of anxiety and CRF for females and not males. Studies suggest that females have higher anxiety sensitivity [53] and a higher CRF level can have a positive effect on anxiety [54] and thus possibly explain the sex difference in this current study. CRF was also associated with body image for all groups except males in 2015. It could be attributable to the fact that the media emphasizes aesthetics rather than performance for males [55], so it is possible that males work out to build muscles rather than to increase CRF. Finally, CRF was only related to self-esteem for females but not for males. At least one other study has shown similar results [56].

It is important to consider the potential impact of SES on the relationship being studied. Prior research has indicated that individuals from lower SES backgrounds are more susceptible to poor mental and physical health outcomes [57,58]. This suggests that the discrepancies in SES between the two groups being compared may have affected the current study’s findings. That is, the 1988 cohort had worse SES than the 1999 cohort. However, if higher SES alone would lead to better mental health, then the 1999 cohort should show better mental health than the 1988 cohort and that was not the case in this current study.

These results highlight the potential benefits of interventions aimed at improving CRF in adolescents, as it may lead to an improvement in mental health. However randomized controlled trials are needed to shed better light on this possible benefit and future research is necessary to identify other potential factors contributing to this relationship and to better understand the underlying mechanisms.

### Differences in mental health depending on the level of cardiorespiratory fitness

The relationship between mental health and the level of CRF is also of importance, as it contributes to the growing body of evidence that suggests that improving CRF levels can possibly have a positive impact on mental health [17,18,59]. In addition, this adds to the converse finding that lower levels of CRF are linked to poorer mental health outcomes [60]. Thus, maximizing CRF levels in adolescents could lead to greater potential mental health benefits. These results highlight the importance of promoting physical activity in this age group and the need for school systems to support physical activity to improve the CRF levels of adolescents [61]. Despite numerous attempts to promote physical activity and fitness among adolescents [62–64], the findings of this current study add to the evidence that those prior efforts have not been effective in reversing the trend of declining mental and physical health in this population. As a result, public health policies could try to incorporate CRF levels when making physical activity recommendations for this age group. That is, put more emphasis on vigorous PA and high levels of CRF to possibly help prevent mental health problems.

### Strength and limitations

The main strength of the study is the proportion of 15-year-olds participating. Since Iceland has a low population, participation reached around 10% of the cohort populations and such percentages in similar studies are rare. The limitation of this study includes the cross-sectional analysis which limits the interpretations of causality. The reliability of the anxiety and body image measures were barley acceptable [65] in 2003 and could be attributable to the fact that few questions were behind the construct [66]. Finally, even though 60% of the population lives in the capital area of Iceland it would’ve made analysis stronger to include other parts of the country in the 2015 data. However, as declared in the method section, no difference was found between variables across location of the participants in the data from 2003. Furthermore, class division is not as apparent in Iceland as it is in many countries. All schools in the study were public and follow the same national curriculum. It can be assumed that these factors increase the likelihood that both cohorts represent the same population. Finally, further research is required to identify the underlying factors contributing to the decline in mental and physical health among adolescents and to understand the mechanisms linking CRF and mental health.

## Conclusions

The findings of this 12-year cross-sectional cohort study suggest that there has been a decline in CRF for adolescents over time. The study also revealed a clear association between CRF and mental health in adolescents, with those who had lower levels of CRF experiencing poorer body image, higher depression and anxiety scores, and lower self-esteem compared to those with higher levels of CRF. Furthermore, the relation between CRF and mental health is more evident for females than males, as highlighted in the regression analysis. However, the ANOVAs also revealed that low levels of CRF increase the likelihood of worse mental health irrespective of the sex of the participant or when it was measured. The findings of this study highlight the possible role of CRF in working on better mental health among adolescents. Therefore, developing effective interventions that focus on improving CRF can be an essential addition to the strategy to promote better mental health in this age group.

## Supporting information

S1 File (CSV)
